# Cross-cultural adaptation and validation of the Italian version of the Kerlan–Jobe Orthopaedic Clinic Shoulder and Elbow score

**DOI:** 10.1007/s10195-017-0467-6

**Published:** 2017-07-14

**Authors:** Giovanni Merolla, Katia Corona, Gustavo Zanoli, Simone Cerciello, Stefano Giannotti, Giuseppe Porcellini

**Affiliations:** 1 Shoulder and Elbow Unit, “D. Cervesi” Hospital, AUSL Della Romagna Ambito Rimini, Via L.V. Beethoven 5, 47841 Cattolica, Italy; 2 Biomechanics Laboratory, “D. Cervesi” Hospital, Cattolica, Italy; 3 Department of Medicine and Health Sciences, University of Molise, Campobasso, Italy; 4 GLOBE, Evidence Based Orthopedics Working Group, Rome, Italy; 5 Casa di Cura Villa Betania, Rome, Italy; 6 Orthopaedic and Trauma Unit, University of Siena, Siena, Italy

**Keywords:** Kerlan–Jobe, Shoulder and elbow, Cross-cultural adaptation, Validation, Italian language

## Abstract

**Background:**

The Kerlan–Jobe Orthopaedic Clinic (KJOC) Shoulder and Elbow score is a reliable and sensitive tool to measure the performance of overhead athletes. The purpose of this study was to carry out a cross-cultural adaptation and validation of the KJOC questionnaire in Italian and to assess its reliability, validity, and responsiveness.

**Materials and methods:**

Ninety professional athletes with a painful shoulder were included in this study and were assigned to the “injury group” (*n* = 32) or the “overuse group” (*n* = 58); 65 were managed conservatively and 25 were treated by arthroscopic surgery. To assess the reliability of the KJOC score, patients were asked to fill in the questionnaire at baseline and after 2 weeks. To test the construct validity, KJOC scores were compared to those obtained with the Italian version of the Disabilities of the Arm, Shoulder, and Hand (DASH) scale, and with the DASH sports/performing arts module. To test KJOC score responsiveness, the follow-up KJOC scores of the participants treated conservatively were compared to those of the patients treated by arthroscopic surgery.

**Results:**

Statistical analysis demonstrated that the KJOC questionnaire is reliable in terms of the single items and the overall score (ICC 0.95–0.99); that it has high construct validity (*r*_s_ = −0.697; *p* < 0.01); and that it is responsive to clinical differences in shoulder function (*p* < 0.0001).

**Conclusions:**

The Italian version of the KJOC Shoulder and Elbow score performed in a similar way to the English version and demonstrated good validity, reliability, and responsiveness after conservative and surgical treatment.

**Level of evidence:**

II.

**Electronic supplementary material:**

The online version of this article (doi:10.1007/s10195-017-0467-6) contains supplementary material, which is available to authorized users.

## Introduction

Overload of an overhead athlete’s shoulder can undermine their sports performance [1–3]. Athletes report shoulder pain and weakness during competition or training sessions, but rarely mention limitations in the activities of daily living. Although several rating systems have been developed to evaluate upper limb clinical outcomes [4], few explore the adverse effects of the repetitive overhead motions that are involved in some athletic movements [5, 6]. The self-administered Kerlan–Jobe Orthopaedic Clinic (KJOC) Shoulder and Elbow questionnaire has been demonstrated to be more reliable and effective in assessing the athletic performance of individuals involved in recreational and professional sports compared to most available tools [7]. The score was developed and validated by Alberta et al. [7], who used the 13-item rating system proposed by Tibone and Bradley [5] as the basis for a new, 10-item subjective evaluation tool. Recent clinical studies have confirmed its superiority in detecting minimal residual functional changes affecting return to play in professional overhead athletes subjected to upper extremity surgery [8–10]. The KJOC Shoulder and Elbow questionnaire was originally developed to assess the functional status of baseball players—which probably accounts for its more widespread use in North America than in Europe, where baseball is less popular—and has subsequently been extended to all overhead sports. The questionnaire has been validated in athletes practicing various overhead sports stratified by injury categories [7]. The purpose of the present study was to perform a cross-cultural adaptation and validation of the 10-item KJOC questionnaire in Italian and to assess its reliability.

## Materials and methods

### Translation and cross-cultural adaptation

Established criteria and guidelines [11, 12] were used for the cross-cultural adaptation and validation of the KJOC Shoulder and Elbow questionnaire in Italian. A professional Italian translator and an Italian orthopedic surgeon each provided an independent Italian translation of the English text of the questionnaire, then two native English speakers performed a new translation of the Italian text into English. The three versions were assessed and merged by a bilingual committee that included clinicians, methodologists, and psychometricians, besides the three translators. The committee ensured that the Italian text took all the relevant Italian cultural features into account, it resolved any conceptual discrepancy by consensus, and agreed on a pre-final text (version 1.0) that was then used in a pilot test involving ten Italian patients. Each patient was asked whether the meaning of each item was clearly understandable, and their impressions were discussed. This work was used to refine the text and to prepare the final Italian version of the questionnaire (version 2.0), which was tested for reliability, validity, and responsiveness in the present study.

### Study population

Professional overhead athletes with a painful shoulder who were evaluated at the Shoulder and Elbow Unit of the “Cervesi” Hospital of Cattolica in Italy from January 2008 to December 2014 offered to participate in this prospective study and gave their informed consent to be included. The study protocol complied with the Ethical Standards of the 1964 Helsinki Declaration as revised in 2000 and was approved by the local ethics committee (Prot. No. 1241/2017 I.5/27). Participants underwent clinical, radiographic, and ultrasound examination of the shoulder before enrollment. They were then asked to complete the 10-item KJOC questionnaire as well as a demographic intake sheet, which included sports participation data and a question asking them if their arm was currently injured. Eleven patients, recruited from January 2008 to March 2010, filled in the original 12-item version of the Kerlan–Jobe Clinic form. These patients were re-called and asked to complete the new validated version by Alberta et al. published on March 24, 2010 [7], and to return it to our Unit by e-mail or fax.

Patients then filled in the Italian version of the Disabilities of the Arm, Shoulder, and Hand (DASH) questionnaire and the DASH sports/performing arts module [13]. This tool was selected for comparison because it is highly reliable and rates the entire upper extremity [14].

As regards cross-cultural adaptation, the description of the level of competition given in the original English version “Please describe your level of competition in your current sport: (use Professional Major League, Professional Minor League, Intercollegiate, High School as the choice)” was translated as “*Descrivi il tuo livello agonistico nel tuo sport attuale: (Professionistico serie maggiori, Professionistico serie minori, dilettante, amatoriale)*” to reflect the Italian league structure and nomenclature. All participants were professional overhead athletes.

Exclusion criteria were previous shoulder surgery, shoulder fractures, intra-articular injections, convulsive disorder, severe comorbidities, cognitive limitations that might have prevented a valid consent or subjective/objective evaluation, and significant shoulder impairment, as reflected by exclusion from competition due to injury. These criteria led us to exclude three patients due to previous shoulder surgery, previous clavicle fracture, and exclusion from competition because of injury, respectively. This left 90 patients, of whom 36 were assigned to the “injury group” (anatomical shoulder injury) (36%) and 58 to the “overuse group” (painful shoulder without injury) (64%).

### Questionnaires

The text of the KJOC questionnaire used in this study was developed and validated by Alberta and colleagues in 2010 [7]. It comprises ten questions asking about the current level of performance and/or function of the involved extremity. Respondents answer by placing a mark on a 10-cm visual analog scale (VAS); its distance from the origin (0 cm) on the left is measured by the examiner to the nearest millimeter and constitutes the score. The scores of the ten questions are summed to obtain a total score that ranges from 0 to 100 (100 = high level of physical capability or “best” score). Questions 5–9 are specific for function and athletic performance and account for 50% of the total score. The rest of the score is provided by questions 1–4, which measure upper extremity symptoms (pain, instability, etc.), and by question 10, which regards the interpersonal relationships related to performance.

The Italian version of the DASH questionnaire [13] was used as a comparison to assess the construct validity of the KJOC Shoulder and Elbow questionnaire.

The DASH questionnaire is a 30-item disability/symptom scale describing the patient’s upper extremity function in the previous week. The questions investigate the amount of difficulty encountered by the patient in performing various physical activities as a result of arm, shoulder, or hand problems (21 items, #1–21); the severity of pain, activity-related pain, tingling, weakness, and stiffness (5 items, #24–28); the influence of these problems on social activities, work, and sleep (3 items, #22, 23, 29); and their psychological impact (1 item, #30). Each item has 5 response options, ranging from “no difficulty or no symptom” to “unable to perform activity or very severe symptom,” and is scored on a 5-point scale. The scores are summed to obtain a scale score ranging from 0 (no disability) to 100 (greatest disability). Participants also completed the DASH sports/performing arts module (also scored from 0 to 100), which includes four items investigating the difficulty encountered by athletes with upper extremity dysfunction in their sports activity.

### Psychometric properties and statistical analysis

Statistical analysis was performed using SPSS 13 for Windows. Descriptive statistics were calculated and reported for all measures. The Shapiro–Wilk test was used to assess the distribution of the data. Median and range were used to describe nonparametric data. Validity, reliability, and responsiveness were investigated.

#### Internal consistency

Internal consistency is the degree of homogeneity of the items within each subscale, and it reflects the correlations between them. It was evaluated using Cronbach’s alpha coefficient. Values ≥0.7 indicate acceptable reliability for scales that are used as research tools to compare groups.

#### Construct validity

Construct validity was measured by assessing the strength of the correlations among the KJOC and the DASH and DASH sports/performing arts module scores, and was evaluated using Spearman’s rank correlation test. Spearman correlation coefficients of >0.50, 0.35–0.50, and <0.35 were considered strong, moderate, and weak, respectively.

#### Reliability

Reliability is the ability of a rating tool to yield the same result and adequate levels of measurement variability upon the repeated administration of the tool in stable subjects. Participants were asked to complete the KJOC questionnaire at baseline and then again 2 weeks later to assess test–retest reliability. The 2-week interval was felt to meet the requirements of minimizing both question recall and changes in the clinical situation or symptom severity. Test–retest reliability was assessed with the interclass correlation coefficient (ICC) for the total score and for the ten items. An ICC of more than 0.80 is considered an indicator of good reliability. Absolute reliability was determined by estimating the standard error of measurement SEM = (SD√1 − ICC) and the minimal detectable difference MDD = 1.96 × √2 × SEM. The mean difference between KJOC test and retest scores plotted against the mean of these two measures is shown as a Bland–Altman plot (95% limits of agreement).

#### Responsiveness

Responsiveness is defined as the tool’s ability to reflect clinically significant changes over time. The responsiveness of the KJOC score to changes in shoulder function in the injury and overuse groups was evaluated by comparing the KJOC score before and after conservative or surgical treatment (6 months) with the Wilcoxon signed-rank test. A value of *p* < 0.05 was considered to be statistically significant. Responsiveness was also assessed by the standardized response mean (SRM) and effect size (ES). The SRM was calculated as the difference between the preoperative mean score and the postoperative mean score divided by the standard deviation (SD) of the difference. The ES was calculated as the difference between the postoperative mean score and the preoperative mean score divided by the preoperative SD.

Median scores or score changes in the injury and overuse group were compared using the nonparametric Kruskal–Wallis test; within-group* p* value changes were computed with the paired Wilcoxon signed-rank test. All tests were two-sided. A value of *p* < 0.05 was considered statistically significant.

#### Discriminant ability

Floor and ceiling effects are defined, respectively, as the percentages with the worst and the best possible scores among all of the patients who completed the questionnaire. Kurtosis and asymmetry indices were also calculated for each item before and after treatment.

#### Feasibility

Feasibility was assessed by considering the time required to fill in the questionnaire, the ease of completion, and the proportion of incomplete questionnaires (with answers missing for >10% of the items).

## Results

The demographic data for the study population are reported in Table 1.

**Table 1:** Demographics of the 90 overhead athletes participating in the study

Variable	Data
Subjects (no.)	90
Age (years) (range)	26.6 (21–34)
Gender (M/F) (%)	51/49 (56)
Height (cm) (SD)	187.8 (7.9)
Weight (kg) (SD)	83.9 (8.6)
Dominant side (no.) (%)	80 (88.9%)
Type of overhead sport (no.) (%)	
Volleyball	66 (73)
Baseball	5 (6)
Swimming	8 (9)
Softball	8 (9)
Tennis	3 (3)

Age, height, and weight data are given as mean values

*SD* standard deviation, *KJOC* Kerlan–Jobe Orthopaedic Clinic, *DASH* Disability of the Arm, Shoulder and Hand

Magnetic resonance scans demonstrated an isolated supraspinatus (SSP) tear in 26 of the 70 athletes who reported severe shoulder pain and weakness during competitions. There were 17 partial tears (10 > 50, 7 < 50%), 9 full-thickness tears, 4 SLAP lesions, and 2 complete SSP tears associated with a SLAP lesion.

### Internal consistency and construct validity

Cronbach’s alpha was 0.910, indicating good homogeneity. Construct validity was assessed by determining the correlation between the KJOC and the DASH scores. Since Shapiro–Wilk’s test showed that the data was not distributed normally (*p* < 0.05), Spearman’s correlation test was used to compare the KJOC and DASH scores (*r*_s_ = −0.697; *p* < 0.01) (Table 2). The correlation was negative, strong, and statistically significant.

**Table 2:** Spearman correlation coefficient between the Kerlan–Jobe Orthopaedic Clinic Shoulder and Elbow score and the Disability of Arm, Shoulder, and Hand score

	DASH	DASH sports/performing arts module
KJOC	−0.697	−0.704

Correlation was significant at the 0.01 level (two tailed)

*KJOC* Kerlan–Jobe Orthopaedic Clinic Shoulder and Elbow score, *DASH* Disability of the Arm, Shoulder, and Hand score

### Test–retest reliability

**Fig. 1:**
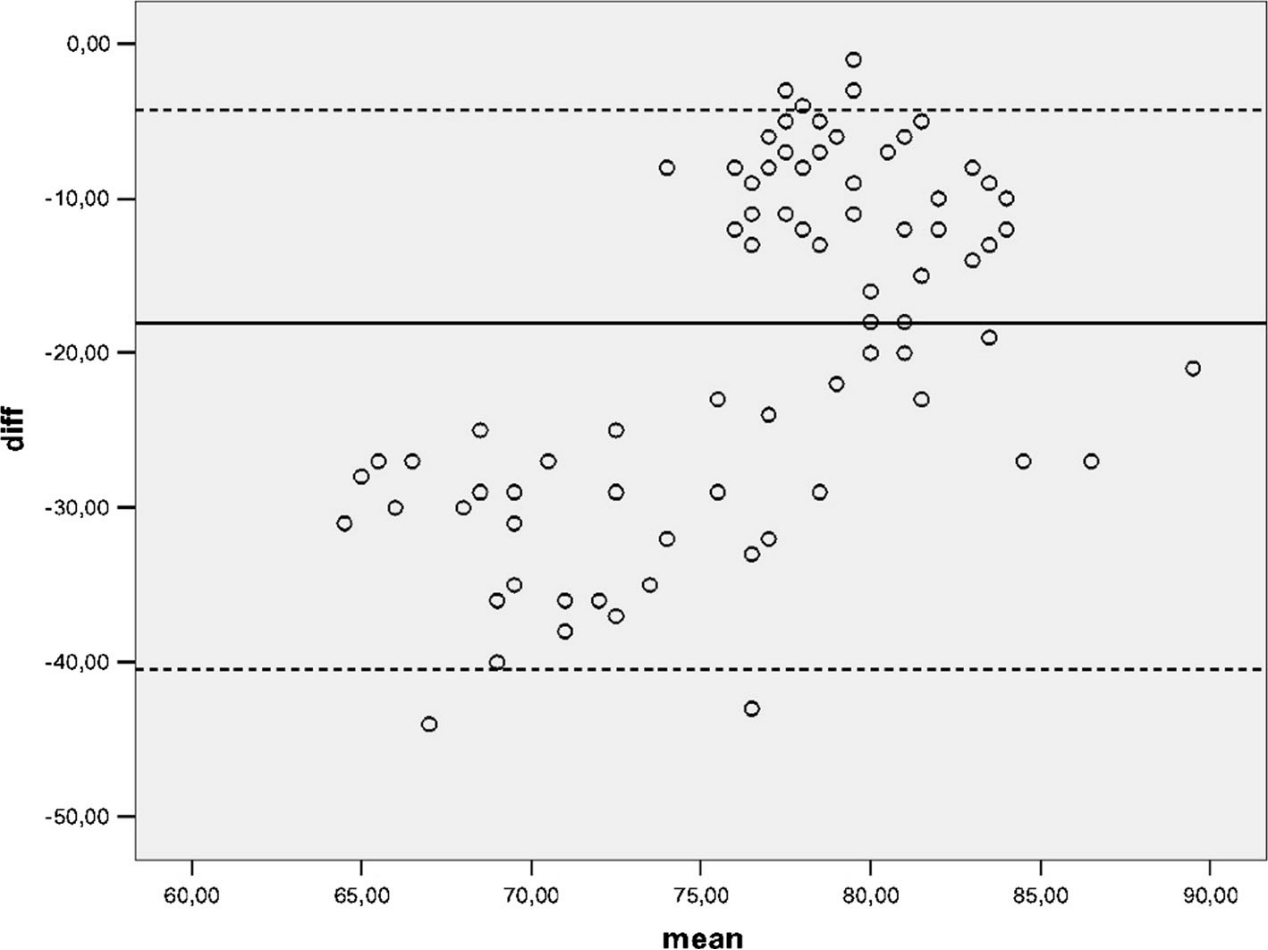
Bland–Altman plot showing the test–retest results of 90 patients who completed the Italian version of the Kerlan–Jobe Orthopaedic Clinic (KJOC) questionnaire. *Solid line* mean difference. *Dashed lines* *upper* and *lower* 95% confidence intervals

The mean value of the KJOC score was 67.84 at the first assessment and 67.92 two weeks later. The ICC of the total KJOC score was 0.99, whereas the ICC for the ten items ranged from 0.95 to 0.99; all values were highly statistical significant (*p* < 0.001) (Table 3). The test–retest reliability of the KJOC questionnaire was high, with an excellent ICC for each item and for the total score. The SEM/MDC was 0.81/2.42, indicating a small measurement error in the screen. The Bland–Altman plot showed a small mean difference (Fig. 1).

**Table 3:** Interclass correlation coefficients (ICC) of the ten items and of the total Kerlan–Jobe Orthopaedic Clinic Shoulder and Elbow score in 90 overhead athletes

KJOC item	ICC^a^
1	0.98
2	0.95
3	0.96
4	0.97
5	0.98
6	0.99
7	0.99
8	0.99
9	0.98
10	0.99
Total KJOC score	0.99

*KJOC* Kerlan–Jobe Orthopaedic Clinic Shoulder and Elbow score

^a^ For all ICC, *p* < 0.0001

### Responsiveness

Twenty-five of the 32 patients in the injury group underwent arthroscopic treatment, whereas 58 patients of the overuse group and 7 of the injury group were managed by a standard 6-month rehabilitation protocol.

The KJOC score was responsive and sensitive to clinical changes in overhead athletes with shoulder dysfunction. The KJOC scores improved significantly after conservative or surgical treatment in both groups, with a median change of 21 points (*p* < 0.0001). ES and SRM were 1.07 and 0.98 in the injury group and 1.34 and 1.27 in the overuse group, respectively (Table 4). A SRM > 0.8 is considered to be excellent. The median change calculated in the injury group (16 points) was significantly lower than the one recorded in the overuse group (32 points) (*p* = 0.0062).

**Table 4:** Responsiveness of the Kerlan–Jobe Orthopaedic Clinic Shoulder and Elbow score in the injury and overuse patients

KJOC score	Injury group (*n* = 32) Pre–post			Overuse group (*n* = 58) Pre–post		
Mean (SD)	56.18 (5.97)	87.43 (5.14)	–	74.27 (2.8)	85.08 (5.02)	–
Median (IQR)	54.5 (52–60)	88 (83.2–90.7)	–	74.5 (72–76)	84 (81–89.2)	–
SEM	1.05	0.90	–	0.37	0.66	–
SRM	–	–	0.98	–	–	1.27
ES	–	–	1.07	–	–	1.34
*p* value	–	–	<0.001	–	–	<0.001

Data refer to the total KJOC scores of injury (*n* = 32) and overuse (*n* = 58) athletes

*KJOC* Kerlan–Jobe Orthopaedic Clinic, *SD* standard deviation, *IQR* interquartile range, *SRM* standardized response mean, *ES* effect size

### Feasibility

The questionnaire was completed without difficulty in an average time of 10 min.

### Discriminant ability

There were no floor or ceiling effects pre- or post-treatment for the total KJOC score.

## Discussion

Patient self-assessment is the most reliable and useful outcome measure in clinical practice, especially where athletic performance and return to competition are concerned; in contrast, the analysis of standard outcome measures—including active mobility, pain, strength, and imaging features—is not ideal, given that professional athletes tend to develop symptoms during competitions or training sessions. In addition, although several upper extremity clinical outcome measures are available to rate high-level athletes, few of them have been developed for the shoulder and elbow [4]. The KJOC Shoulder and Elbow questionnaire is a validated clinical outcome tool that provides highly valuable information on the performance of overhead athletes compared to existing traditional shoulder and elbow rating instruments [7]. Moreover, it is easy to use and score and is equally effective in evaluating the shoulder and the elbow. The Conway-Jobe scale, which has been developed as an outcome tool to rate the athlete’s functional status following injury, has in fact been applied only to the elbow and has not been validated [6].

Like most upper extremity rating systems, the KJOC Shoulder and Elbow questionnaire was developed for English speakers. This means that modifying it for use by speakers of other languages requires translation as well as cross-cultural adaptation, which together ensure the cultural equivalence of the texts in the two languages [15]. Moreover, culturally equivalent outcome measures make assessments performed in different countries more reliable, they simplify the meta-analysis for clinical research, and they reduce the risk of bias [15, 16].

In this study, the Italian version of the KJOC Shoulder and Elbow questionnaire was applied to test Italian patients using a systematic and standardized approach [11, 12]. The questionnaire proved easy to complete in a reasonably short time. No significant difficulties were encountered when translating it from English to Italian, except for one question where the fact that the level of competition in the original text is described in relation to the American league system required adaptation to Italian professional sport categories.

The strong correlation of the KJOC Shoulder and Elbow score with the DASH and the DASH sports/performing arts module score supports the validity of the KJOC tool. A Cronbach alpha of 0.91 indicated high internal consistency, while the ICC values for the ten items and for the total score (0.95–0.99) indicated excellent reliability, similar to that for the published English version. The lack of ceiling and floor effects confirms the validity of the Italian version of the KJOC questionnaire.

An additional factor addressed in the validation of the KJOC questionnaire involved the quantification of clinical differences between the athletes with anatomical and overuse injuries. Interestingly, the median KJOC score change from baseline following conservative or surgical treatment was significantly lower in the injured athletes than in those without lesions.

This is the first study describing the validation and cross-cultural adaptation of a clinical questionnaire rating overhead athletes in a language other than English. The questionnaire items provide an excellent and sensitive approach to assess the ability of professional overhead athletes to return to the same level of competition as before shoulder and elbow injury. The present study has some limitations. First of all, the statistical power was not calculated ab initio, but a population size comparable to those used in similar studies was recruited to avoid major biases. In addition, most overhead athletes were volleyball players (as noted above, some sports, such as baseball, are played less in Italy than in the United States). Finally, the study is based on a prospective analysis of a population that had already been selected and scheduled for conservative or surgical treatment in relation to the type of shoulder injury. Despite these limitations, the Italian version of the KJOC questionnaire demonstrated good psychometric properties that were comparable to those of the original questionnaire. We believe that the Italian version of the questionnaire can be used to evaluate outcomes in Italian-speaking patients with shoulder dysfunction, and that it provides a sound basis for future clinical trials involving similar populations.

In conclusion, the Italian version of the KJOC Shoulder and Elbow score performed in a similar way to the English version and proved easy to complete in a reasonably short time. It demonstrated high internal consistency, excellent reliability (similar to that of the published English version), and good responsiveness after conservative and surgical treatment.

## Electronic supplementary material

Below is the link to the electronic supplementary material.

Supplementary material 1 (DOCX 140 kb)
