# Supplementary material for: Cross-cultural adaptation and validation of the Italian version of the Kerlan–Jobe Orthopaedic Clinic Shoulder and Elbow score
Source: J Orthop Traumatol. 2017 Jul 14;18(4):415–21. doi: 10.1007/s10195-017-0467-6 (PMC5685985; doi:10.1007/s10195-017-0467-6)
Supplement: Supplementary file 1 — Supplementary material 1 (DOCX 140 kb) [file 10195_2017_467_MOESM1_ESM.docx]

**Punteggio di Spalla e Gomito della Clinica Ortopedica Kerlan-Jobe**

Nome_____________________ età__________ sesso________ data di valutazione_________________

Arto dominante (D)_______ (S)_________ (Ambidestro)___________

Sport____________________ Ruolo_________________ Anni di attività ____________

Ti chiediamo cortesemente di rispondere alle seguenti domande relative **solo alla tua anamnesi di lesioni al braccio:**

1. Il tuo braccio è attualmente infortunato? ☐si ☐ no
2. Stai giocando o praticando attivamente il tuo sport? ☐si ☐ no
3. Nel corso dell’ultimo anno ha mai saltato gare o allenamenti per una lesione alla spalla o al braccio? ☐si ☐ no
4. Ti è stata diagnosticato uno strappo o distorsione alla spalla o al braccio? ☐si ☐ no

In caso di risposta affermativa quel’è stata la diagnosi? __________________________________

1. Sei stato curato per una lesione della spalla o del braccio? ☐si ☐ no

Se si qual’ è stato il trattamento? (è consentito dare più di una risposta):

☐riposo ☐ terapia ☐ intervento chirurgico

**Ti chiediamo cortesemente di descrivere il tuo livello agonistico nel tuo attuale sport:**

(☐ professionistico serie maggiori ☐Professionistico serie minori ☐Livello scolastico nazionale ☐Scolastico locale)

1. Qual è il livello agonistico più alto che hai raggiunto?____________________________
2. Qual’ è il tuo attuale livello agonistico?___________________________________
3. Se il tuo livello agonistico attuale non è quello più alto che hai raggiunto, credi che sia dovuto ad una lesione del tuo braccio? ☐si ☐ no

Ti chiediamo cortesemente di barrare la risposta che descrive al meglio il tuo attuale stato:

☐Gioco senza alcun problema al braccio ☐Gioco ma con problema al braccio

☐Non gioco a causa di problemi al braccio

**Istruzioni per gli atleti:**

Le seguenti domande riguardano la tua condizione fisica durante la gara e in allenamento. A meno che non sia diversamente specificato, tutte le domande riguardano la tua spalla e gomito. Ti chiediamo cortesemente di rispondere con una X lungo la linea orizzontale che corrisponde al tuo attuale livello.

1. Quanto è difficile rilassarti o riscaldarti prima della competizione o dell’allenamento?

Non mi sento mai rilassato Normale tempo di riscaldamento

in gara o in allenamento

1. Quanto dolore hai sentito nella tua spalla o gomito?

Dolore a riposo Nessun dolore durante la competizione

1. Quanto debolezza o fatica (i.e, perdita di forza) hai provato nella tua spalla o gomito?

Debolezza o fatica che mi ha precluso Nessuna debolezza, normale fatica in competizione

ogni competizione

1. Quanto instabile senti la tua spalla durante la competizione?

“Salta fuori” abitualmente No instabilità

1. I fastidi al tuo braccio quanto fanno influenzato i rapporti con il tuo allenatore, la tua amministrazione e i tuoi agenti?

Ho lasciato la squadra,

sono stato ceduto, oggetto di rinuncia, Niente affatto

ho perso il contratto o la borsa di studio

Le seguenti domande si riferiscono al tuo livello agonistico nel tuo attuale sport. Ti chiediamo cortesemente di rispondere con una X lungo la linea orizzontale che corrisponde al tuo attuale livello.

1. In che misura ha dovuto modificare il tuo movimento nel lancio, servizio, battuta, ecc a causa del tuo braccio?

Cambiamento completo, Nessun cambiamento nel movimento

Non eseguo più quel movimento

1. Che limiti ha la tua velocità o potenza a causa del tuo braccio?

Ho perso tutta la forza , nessun cambiamento in velocità/potenza

MI sono dedicato a gare di abilità o sulla distanza

1. Che limiti di resistenza in partita hai a causa del tuo braccio?

Notevole limitazione Nessuna limitazione nella resistenza in competizione

(per es. sono diventato una riserva,

sono passato a gare brevi)

1. In che misura il tuo controllo (lanci, servizi, battute, ecc) ha sofferto a causa del tuo braccio?

Controllo imprevedibile di tutti i lanci, Nessuna perdita di controlllo

servizi, battute, ecc

1. In che misura il tuo braccio influenza il tuo livello agonistico nel tuo sport (cioè il tuo braccio ti ti impedisce di essere al massimo del tuo potenziale)

Non posso fare attività agonistica, gioco al livello agonistico desiderato,

ho dovuto cambiare sport
